# Case of Double Monster—Morbid States of the Placenta

**Published:** 1853-07

**Authors:** Wm. F. Montgomery

**Affiliations:** Dublin


*Case of Double Monster—Morbid states of the Placenta.* By Wm. F. Montgomery, A. M., M. D., etc., Dublin.—March 6th, 1851, Mr. O'Donovan was called to Mrs. L., aged 35, mother of three children, and seven months pregnant of her fourth, in very delicate health; her abdomen enormously enlarged, having the appearance of a twin pregnancy in the ninth month; lower extremities œdematous; and she was exceedingly weak and dispirited; former pregnancies favorable.

11 o'clock, P. M., labor set in rapidly. Mr. O'Donovan found the membranes ruptured and the room deluged with the waters; the feet and legs of a child were protruded, cold and livid; at the orifice of the vagina appeared what at first seemed a hand, but being pushed down it was found to be a third leg, then a fourth leg was discovered and brought down.

By passing the finger round the fœtus, Mr. O'Donovan found that the body was single at the umbilicus, the cord lying in the cleft, and a portion of intestine protruding. He assisted the pains, which were few and weak, gradually drew down the body, and, having hooked down the arms, delivered the woman of a full-grown monster fœtus, with two heads and two sets of extremities; the placenta soon descended and was removed; the heads passed one after the other, moving, as it were, on a central point, the junction at the neck. There was imperfect contraction of the womb, and a tendency to hemorrhage, which, however, Mr. O'Donovan was enabled to prevent by the usual means, and the mother ultimately did well.

"No description," says Mr. O'Donovan, "can convey an idea of the horrible appearance of this monster: the heads taken together were much larger than the head of a full-grown healthy child, and were fully developed; the faces fronting and applied to each other, the mouth of the one being received into that of the other, which was a large chasm, the line of junction commencing at the lower lip and continuing perfect to the umbilicus; the thorax of each was well formed and distinct except for the juncture; at the dorsal vertebræ, and opposite the umbilicus, the bodies were drawn towards each other as if compressed by a cord. The gross bulk of the double monster was equal to that of a single child of nine months; each head was as fully ossified as a natural fœtus of the same age, that is, seven months. The abdominal parietes were deficient at the junction of the funis, where there existed only a thin diaphanous membrane, as if an expansion of the sheath of the cord itself; the arms of the children embrace each other as in the form of a figure of 8. The placenta was about the natural size, part apparently healthy, but nearly a third *presented a vast collection of hydatids.* No examination would be permitted."

Mr. O'Donovan has lately informed me that the lady was again confined last month (February), under his care, of a healthy, full grown, female child, and had a most favorable labor.

I may observe, in reference to this case, how often anomalous or morbid states of the cord or placenta are, as indeed we might anticipate, found in connection with malformations or monstrosities of the fœtus; hydatid degeneration of the placenta is often met with, not only accompanied by extreme atrophy of the fœtus, but frequently no trace of it is left remaining.

One of the ugliest and most disgusting-looking monsters I ever saw was connected with an enormous placenta, whose substance was quite unravelled, and the separate branches of capillaries hung down like minute stalactites, at least two inches long, while the twin brother of this monster was a comely child, attached to a healthy placenta and cord.

Such a relation between morbid anomalies of the placenta and malformations or monstrosities in the fœtus must cease to surprise us when we consider that the placenta is, in fact, the organ or apparatus by which the whole *pabulum vitæ* is supplied for the development and support of the child during its intra-uterine life, and if this supply is tainted at its source, or interrupted by morbid changes therein, we can readily understand to what a degree the evolution of the embryo is likely to be thereby affected.

Hence it is, that in those perplexing cases so frequently met with, in which women are, in several successive pregnancies, delivered of dead children, without any discoverable constitutional disease or infirmity in the mother, an examination of the placenta so frequently discloses to us morbid alterations quite sufficient to explain the distressing result. Of these I may just allude to what has been called tubercular disease of the placenta, apoplexy of its substance, hydatid degeneration, inflammation of the placenta or envelopes of the ovum, and especially if producing their adhesion to the surface of the child.

In an other instance under my observation, the cord was excessively dropsical, so as to be in some parts two inches in diameter, and in that case the child was unequally developed, one side of the body being very much larger than the other; this inequality, however, gradually disappeared, and the young lady is now of unexceptionable symmetry. "In one of the recorded cases," says Vrolik, "the superior extremities were wanting, and the anus was closed."

Serres considers the absence of one of the umbilical arteries as the efficient cause of that form of monstrosity in which there is *ectopia viscerum abdominalium*, but such a consequence does not always follow such a defect. I have in my museum a specimen of a placenta and umbilical cord with only one artery, and it was connected with a remarkably fine, healthy, and well-formed child.

This want of one umbilical artery was also observed in Sir A. Cooper's imperfect and heartless fœtus, but, with the exception of an umbilical hernia, there was no other *ectopia viscerum abdominalium.*

I have for many years endeavored to impress on my pupils the great advantages to be gained not only in the way of general pathological inquiry, but in the acquisition of a particular species of knowledge of the utmost practical value, by carefully examining the fœtus and its envelopes in every case, but especially in those cases of blight or arrested development, where the size of the fœtus is so entirely at variance with the real date of the pregnancy, a mistake on which point may lead, and has often led, to giving an opinion which may irreparably injure a really unblemished reputation.

The following case, which was recently brought under my observation, is a striking illustration of the above remark. Five months after her husband's departure for a foreign country, a lady miscarried of an ovum and fœtus, presenting the characters and development of the third month. For more than two months and a half after the separation of the parties she had no menstruation, and had other indications of pregnancy; but she then had sanguineous discharges from the vagina, which were regarded as a return of her catamenia, and she was no longer considered pregnant. These, however, ceased, and there was again a suppression for two months and a half, at the end of which time the lady miscarried of an ovum and fœtus, presenting conditions corresponding to such a period; the result of which was a conviction, on the part of some members of her husband's family, that she must have been unfaithful to him, and it was at once decided to inform him of his misfortune. Before doing so, however, the ovum was shown to a medical friend, who, wishing to have his own judgment in so delicate a matter fortified by the opinion of another, submitted the ovum to me for careful examination, when the true nature of the case appeared at once manifest: the envelopes were in a morbid state, thickened and tuberculated, and had evidently been long separated from the vascular connection with the uterus; the umbilical cord also was diseased at its placental end, where it was expanded into a lotus-shaped sac, filled with a brownish serum. Of the true history of the case there seemed, then, no doubt; the lady had conceived just at the time of her husband's departure, her pregnancy had proceeded undisturbed until the third month, when she had symptoms of miscarriage, but did not miscarry; but the ovum was blighted, and, having lost its vitality, ceased to grow; it was, however, retained in the uterus until the expiration of five months from the date of conception, when it was expelled in the morbid state already described. This explanation at once set at rest all the unworthy and undeserved suspicions entertained against this innocent lady, who would otherwise have been made the subject of a most painful proceeding. Facts of this kind have been heretofore insisted on by the writer in his work on the Signs of Pregnancy, and some cases related in illustration.—*Boston Med. and Surg. Jour.*—*Dublin Quar. Med. Jour.*

